# MaHSFA2c modulates high temperature-inhibited chlorophyll breakdown in banana fruit by directly inducing the transcription of the E3 ligase gene *MaBAH1*

**DOI:** 10.1186/s43897-025-00209-4

**Published:** 2026-04-03

**Authors:** Wei Wei, Meng-xue Liu, Jia Si, Qi Luo, Yumei Zhang, Jian-fei Kuang, Jian-ye Chen, Wang-jin Lu, Wei Shan

**Affiliations:** State Key Laboratory for Conservation and Utilization of Subtropical Agro-bioresources/Guangdong Provincial Key Laboratory of Postharvest Science of Fruit and Vegetables/Engineering Research Center of Southern Horticultural Products Preservation, Ministry of Education, College of Horticulture, South China Agricultural University, Guangzhou, 510642 China

**Keywords:** Banana fruit, Green ripening, HSF, Chlorophyll catabolism, High temperature

## Abstract

Bananas (*Musa acuminata*, AAA group) ripening at temperatures higher than 24 °C prevent yellow skin development, resulting in green ripening, and significantly affecting the commercial potential of the fruit. Our recent study discovered that high temperature (HT) triggers the E3 ubiquitin ligase MaBAH1 to degrade MaMYB60, thereby suppressing MaMYB60's transactivation of chlorophyll catabolic genes. However, the mechanism by which HT stimulates *MaBAH1* expression remains unknown. In this study, a heat shock transcription factor, termed MaHSFA2c, was identified as an upstream regulator of *MaBAH1*. MaHSFA2c gene expression and protein levels were up-regulated in green-ripened fruit at 30 °C compared to yellow-ripened fruit at 20 °C. MaHSFA2c directly activated the transcription of *MaBAH1*. Importantly, silencing *MaHSFA2c* in banana fruit suppressed *MaBAH1* expression, reducing MaBAH1-mediated MaMYB60 degradation. This promoted MaMYB60's activation of chlorophyll degradation, thereby weakening green-ripening at HT. In the context of banana fruit, we have demonstrated the existence of a dynamic regulatory module consisting of MaHSFA2c and MaBAH1 that manages chlorophyll catabolism inhibition under HT. These findings enhance our understanding of the transcriptional regulation involved in the loss of fruit quality due to temperature stress and are expected to aid in developing strategies to manage banana green-ripening.

**Supplementary Information:**

The online version contains supplementary material available at 10.1186/s43897-025-00209-4.

## Core

MaHSFA2c is activated when bananas ripen at high temperatures. MaHSFA2c activates *MaBAH1* transcription, leading to MaMYB60 degradation and subsequent inhibition of CCG expression. Consequently, chlorophyll degradation is inhibited, preventing banana fruit from developing a yellow peel and causing them to ripen while remaining green.

## Gene & accession numbers

Squence data can be found in the banana genome database (https://banana-genome-hub.southgreen.fr/) under the following accession numbers: MaHSFA2c (Ma05_g20130), MaBAH1 (Ma05_g02600), MaMYB60 (Ma04_g00460.1).

## Introduction

In a global context, bananas (*Musa acuminata*, AAA group) are one of the mostfrequently consumed fresh fruits, primarily grown in developing countries due to their specific climatic requirements. They also serve as a major multibillion-dollar export commodity for developed nations. Typically, bananas are harvested at the mature-green stage and then transported to wholesale markets, where they ripen to a golden yellow using ethylene (Wu et al. 2023). During the postharvest ripening period, temperature is a pivotal factor affecting banana fruit quality. Ripening at temperatures exceeding 24 °C, such as 30 °C, accelerates softening while inhibiting chlorophyll breakdown, resulting in a green ripening phenotype (Wei et al. 2023a). This green ripening is an unfavorable and unprofitable condition that negatively impacts banana quality in the market. Recently, global warming and climate change have led to high temperature (HT), causing green ripening and significantly threatening both banana fruit quality and the economic viability of the banana industry. The suppressed chlorophyll breakdown in bananas at 30 °C represents an atypical response to HT when compared to other fruits like tomatoes and mangoes, where chlorophyll breakdown occurs more rapidly at the same temperature than at lower temperatures (Ogura et al. 1975; Medlicott et al. 1986). Additionally unlike pears and avocados, which discolor at temperatures up to 40 °C (Maxie et al. 1974; Eaks 1978), the minimum temperature of 24 °C required to prevent banana degreening is theoretically insufficient to inhibit enzymatic reactions (Yang et al. 2009). Therefore, understanding the mechanisms behind HT-induced color deterioration in bananas is crucial for developing strategies to control green ripening.

Fruit color regulation is a complex biological process that involves a combination of various regulators. Studies conducted during the last few years revealed that numerous transcription factors regulate pathways closely related to fruit pigment synthesis, such as the chlorophyll, carotenoid, and anthocyanins metabolic pathways (Espley And Jaakola 2023; Muhammad et al. 2024). For example, apple transcription factors MdMYB10, MdWRKY11, MdNAC1, and MdbZIP23 are responsible for flesh color in red-fleshed cultivars through activation of the structural anthocyanin synthesis genes (Rihani et al. 2017; Liu et al. 2019; 2023). Tomato non-ripening like 1 (NOR-like 1) directly activates chlorophyll catabolic gene STAY-GREEN 1 (SlSGR1), and NOR-like knockout fruit presents an orange phenotype at the red ripening stage, suggesting that NOR-like1 is an important regulator in fruit color development (Gao et al. 2018). In citrus, CrWRKY42 and CsMADS3 coordinately regulate chlorophyll degradation and carotenoid biosynthesis to alter the conversion of fruit color by activating the genes involved in both pathways (Zhu et al. 2023; Chen et al. 2024). Environmental stress commonly regulates the activity of transcription factors via various post-translational modifications, such as phosphorylation along with ubiquitylation (Ding et al. 2020). Low temperatures suppress the E3 ligase MdMIEL1, preventing the degradation of MdMYB308, which in turn enhances the transactivation of anthocyanin biosynthesis genes by MdMYB308, leading to increased anthocyanin accumulation in apples (An et al. 2020). Conversely, in strawberry fruits, low-temperature stress increases FvMAPK3 (MITOGEN-ACTIVATED PROTEIN KINASE3) activity, which in turn, enhances the phosphorylation and reduces the transcriptional activity of FvMYB10, a central activator of anthocyanin biosynthesis pathway, and eventually leads to the repression of anthocyanin accumulation and poor coloration (Mao et al. 2022). However, compared with understanding how post-translational modifications affect TFs' activity, little is known about how TFs' modifications are triggered by environmental stress, especially how their post-translational modifiers are regulated.

Heat shock transcription factors (HSFs) are crucial in HT signal transduction, significantly contributing to thermotolerance and the heat stress response in plants (Zhao et al. 2022; Shekhawat et al. 2022). HSFs possess a conserved structure featuring an N-terminal DNA binding domain (DBD) that primarily identifies heat shock cis-elements in target promoters. Additionally, they have an adjacent oligomerization domain HR-A/B, which is formed of hydrophobic heptad repeats, which facilitates multimer formation (Muthuramalingam et al. 2020). HSFs can be categorized into HSFA, HSFB, and HSFC classes based on the HR-A/B domain structure (Guo et al. 2016). HSFA members generally serve as transcriptional activators, whereas HSFB and HSFC members function as transcriptional repressors or co-activators (Andrási et al. 2021). HSFs constitute a sophisticated transcriptional regulatory cascade to cope with heat stress situations. For instance, Arabidopsis AtHSFB1/2b represses the expression of heat-inducible HSF genes, such as HSFA2/7a and HSP genes (Ikeda et al. 2011). AtHSFA2 binds AtHSFA3 to form heteromeric complexes with other HSFAs, which in turn promotes transcriptional memory after heat stress (Friedrich et al. 2021). AtHSFA1s interact and cooperate with HSFA2 to activate genes encoding HS-responsive proteins (Shekhawat et al. 2022). Currently, HSF function in fruit quality and tolerance to abiotic stress in economically important fruit crops has attracted much attention. EjHSF1 and EjAP2 collaboratively regulate lignin biosynthesis in loquat fruit under chilling stress (Zeng et al. 2016), while CitHsfA7 directly activates the citrate metabolism gene to manage heat-induced citrate degradation in citrus fruit (Li et al. 2022). The latest research found that banana MaHsf24 negatively regulates fruit cold tolerance via suppressing genes encoding HSPs and antioxidant enzymes (Si et al. 2024). Nonetheless, the connection between HSFs and fruit deterioration due to environmental stress remains largely unexplored.

An E3 ligase, MaBAH1 (benzoic acid hypersensitive 1), was recently identified from banana fruit (Wei et al. 2023a). When the banana was ripened at HT, MaBAH1 is induced and degrades the MYB transcription factor MaMYB60, which inhibits MaMYB60-induced transactivation of chlorophyll catabolic genes and eventually suppresses chlorophyll degradation (Wei et al. 2023a). However, how MaBAH1 is regulated in response to HT is completely unclear. Herein, an HSF family member, MaHSFA2c, was identified as the potential binding protein for the MaBAH1 promoter. MaHSFA2c gene expression and protein levels are higher in green-ripened fruit at 30 °C than in yellow-ripened fruit at 20 °C. MaHSFA2c displayed transcriptional activation activity and activated the transcription of *MaBAH1*. Silencing *MaHSFA2c* in banana fruit suppressed *MaBAH1* expression, reducing MaBAH1-mediated MaMYB60 degradation and promoting MaMYB60's activation of chlorophyll degradation, thereby weakening green-ripening at HT. The study identifies a novel regulatory module, MaHSFA2c-MaBAH1, which controls HT-inhibited chlorophyll degradation in banana fruit, enhancing the understanding of transcriptional regulation of fruit quality deterioration due to temperature stress.

## Results

### MaHSFA2c directly targets the *MaBAH1* promoter

**Fig. 1:**
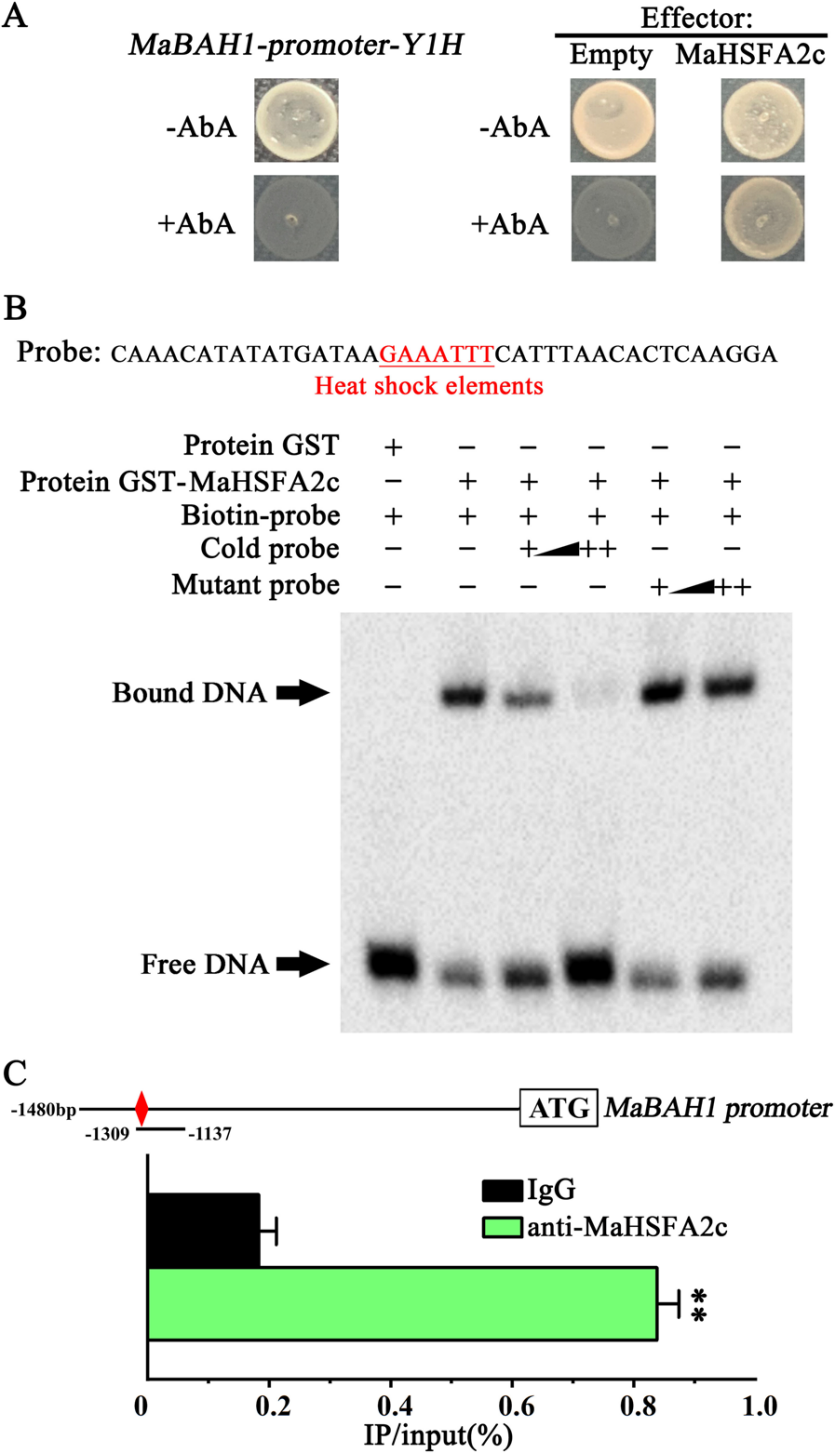
MaHSFA2c binding to the *MaBAH1* promoter. **A** Y1H analysis examining the interaction between MaHSFA2c and the *MaBAH1* promoter. Culture of reporter yeast strains transformed with MaHSFA2c effector or empty vector on SD-Leu medium with 500 ng/mL aureobasidin A. No basal promoter activity was observed. **B** EMSA of in vitro binding of MaHSFA2c to the *MaBAH1* promoter. Triangles denote overexpressed unlabeled WT or mutant probes for competition. **C** ChIP-qPCR assay of in vivo binding of MaHSFA2c to the *MaBAH1* promoter. A schematic of the promoter structure highlights HSEs (red diamonds) and probe regions (black underlines). Data represent mean ± SE (*n* = 3); Significance: *p* < 0.01, Student's *t*-test

Our prior research indicated that elevated temperatures impede chlorophyll breakdown in banana fruits by promoting MaBAH1-mediated degradation of MaMYB60 (Wei et al. 2023a). To obtain an understand the regulatory mechanisms by which HT induces *MaBAH1* expression, the *MaBAH1* promoter was isolated, and its interacting proteins were identified through yeast one-hybrid assay (Y1H) screening using a cDNA library from banana peels. After screening, a cDNA fragment encoding a peptide from the HSF family member (Ma05_p20130) was identified. Since Ma05_p20130 has the closest evolutionary relationship with rice OsHSFA2c (Fig. S1A), it was designated as MaHSFA2c. The one-to-one Y1H assay was employed to confirm the interaction between the MaHSFA2c protein and the MaBAH1 promoter, using the full coding DNA sequence of *MaHSFA2c* as the prey. The *MaBAH1* promoter showed no basal activity in yeast when exposed to the yeast toxin AbA (Fig. 1A). Co-expression of *MaHSFA2c* enhanced AbA resistance gene expression under the control of the *MaBAH1* promoter, enabling transformed yeast to grow robustly on AbA-supplemented medium. This indicates that MaHSFA2c binds to the *MaBAH1* promoter.

Amino acid sequence alignment showed that MaHSFA2c comprises a conserved HSF-type DNA binding domain (Fig. S1B), which can recognize HSEs in the target gene promoters (Bienz And Pelham 1986). Analysis of the nucleotide sequence identified HSEs within the MaBAH1 promoter (Fig. S2). To assess the direct binding capability of MaHSFA2c to the *MaBAH1* promoter, an in vitro electrophoretic mobility shift assay (EMSA) was conducted. Figure 1B presents the purified recombinant GST-MaHSFA2c (Fig. S3), which, unlike GST alone, directly bound to DNA fragments with HSEs from the *MaBAH1* promoter, causing noticeable mobility shifts. The introduction of increasing quantities of unlabeled probes sharing an identical sequence markedly diminished band shifting, while modified competitors failed to achieve this outcome (Fig. 1B). In vivo chromatin immunoprecipitation (ChIP)-qPCR showed that the anti-MaHSFA2c antibody significantly precipitated the promoter regions containing HSEs of MaBAH1 compared with the negative control (nonspecific IgG antibody) (Fig. 1C). Collectively, these findings indicate that MaHSFA2c exclusively interacts with the *MaBAH1* promoter.

### HT suppresses chlorophyll breakdown and promotes MaHSFA2c gene expression and protein accumulation in banana fruit

**Fig. 2:**
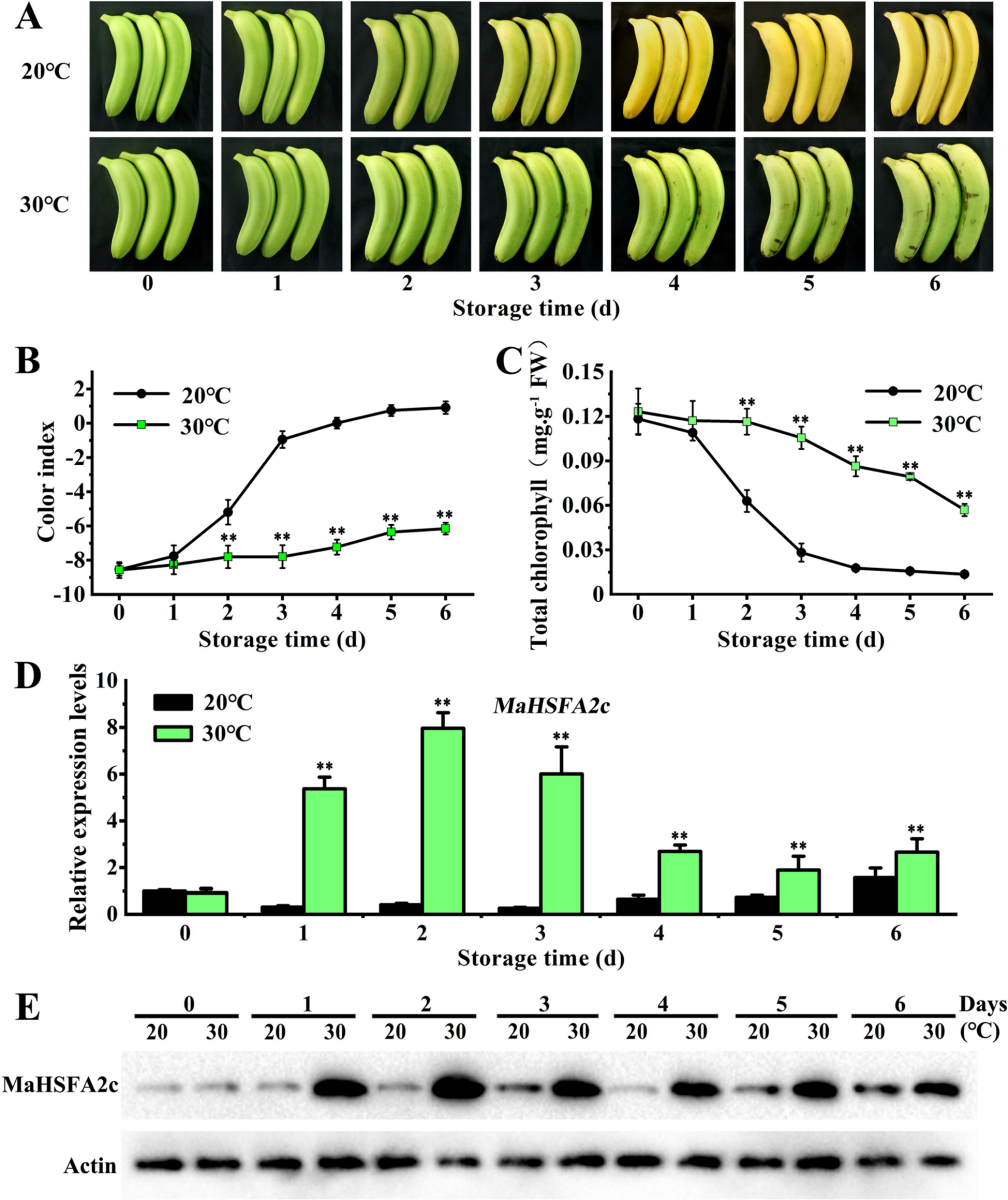
MaHSFA2c transcript and protein dynamics in banana fruit during ripening at 20 °C and 30 °C. **A** Visual changes in banana peel appearance throughout ripening at 20 °C and 30 °C. **B**-**C** Variations in peel color index (**B**) and total chlorophyll content (**C**) over the ripening period. **D** *MaHSFA2c* transcript levels throughout ripening at 20 °C and 30 °C, expressed as fold changes relative to day 0 (set as 1). E MaHSFA2c protein accumulation during ripening, with actin serving as the loading control. Error bars represent SE (*n* = 6 for (**B**) and (**C**); *n* = 3 for (**D**)). Asterisks: Significance (** *p* < 0.01, Student's *t*-test)

Banana fruits ripened at 20 °C exhibited yellowing by day 3 and were fully de-greened by day 5. In contrast, those ripened at 30 °C showed no significant yellowing even by day 6, indicating green ripening (Fig. 2A). Consistently, fruits ripened at 20 °C exhibited a more pronounced increase in CI and a quicker decline in total chlorophyll content compared to those ripened at 30 °C (Figs. 2B–C). RT-qPCR and Western blot analyses were used to measure MaHSFA2c transcript and protein levels in banana peels throughout ripening at 20 °C and 30 °C to explore its potential link to green ripening. The transcript level of *MaHSFA2c* was significantly higher in fruit ripened at 30 °C, compared to 20 °C throughout the ripening period (Fig. 2D). Similarly, exposure to 30 °C, in comparison to 20 °C, significantly increased MaHSFA2c protein accumulation during the ripening process (Fig. 2E). Collectively, MaHSFA2c transcript induction and subsequent protein accumulation under HT is related to the HT-suppressed chlorophyll degradation in bananas.

### Molecular characterization of MaHSFA2c

**Fig. 3:**
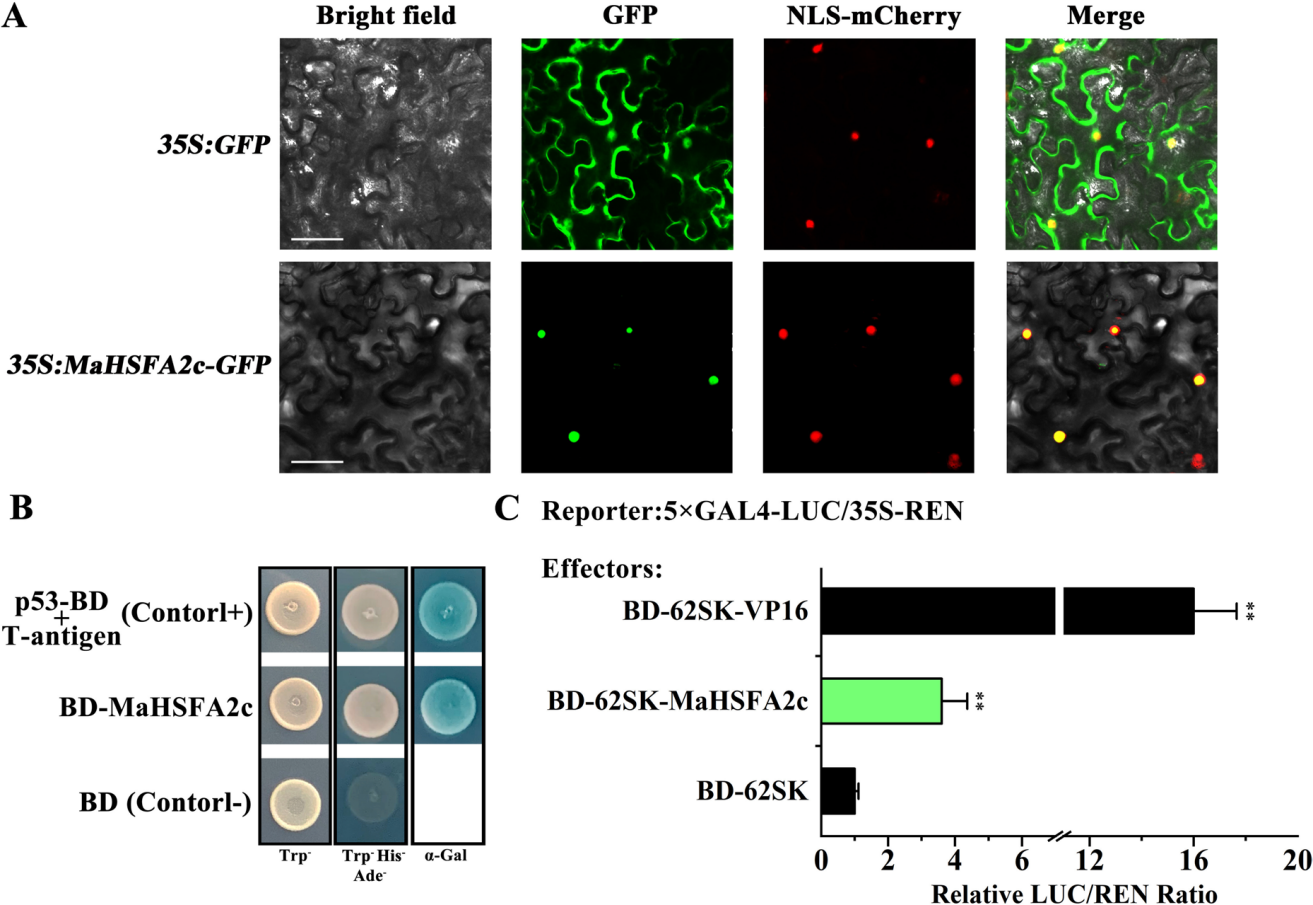
MaHSFA2c molecular characterization. **A** MaHSFA2c subcellular localization in *Nicotiana benthamiana* leaves. Transient expression of MaHSFA2c-GFP fusion protein and GFP (control) via agro-infiltration. Scale bars: 50 µm. **B** MaHSFA2c transcriptional activity in yeast cells. Culture of yeast transformed with various plasmids on SD media lacking tryptophan, or tryptophan, histidine, and adenine. Yeast growth and α-galactosidase activity: transcriptional activation. **C** MaHSFA2c trans-activation capacity in tobacco leaves. Calculation of LUC/REN ratio, setting the empty BD-62SK vector (negative control) to 1 and BD-62SK-VP16 as the positive control. Data represent mean ± SE (*n* = 6); Asterisks: Significance (*p* < 0.01; Student's *t*-test)

To examine the subcellular localization of MaHSFA2c, we fused it with GFP and transiently expressed the fusion in tobacco leaves. Co-expression of NLS-mCherry allowed us to monitor the nucleus. The GFP signals from MaHSFA2c-GFP combined with the RFP signal from NLS-mCHerry, indicating the nuclear localization of MaHSFA2c (Fig. 3A).

The transcriptional capacity of MaHSFA2c was evaluated using a GAL4-responsive reporter system in both yeast and tobacco leaf cells. The Y2HGold yeast cells transformed with DBD-MaHSFA2c, along with the positive control, survived in a selective medium lacking Trp, His, and Ade and exhibited X-α-Gal activity (Fig. 3B). In contrast, yeast cells transformed with the negative control did not grow, implying that MaHSFA2c possesses transcriptional autoactivation in yeast. To verify these results, a dual-LUC reporter assay was performed in tobacco leaves. This assay involved connecting the LUC reporter to a TATA box and five GAL4 DNA-binding elements. As shown in Fig. 3C, similar to the transcriptional activator VP16, MaHSFA2c significantly increased the LUC/REN ratio relative to the empty BD vector, demonstrating its transcriptional activation activity in planta.

### MaHSFA2c induces the expression of MaBAH1 and inhibits chlorophyll degradation

**Fig. 4:**
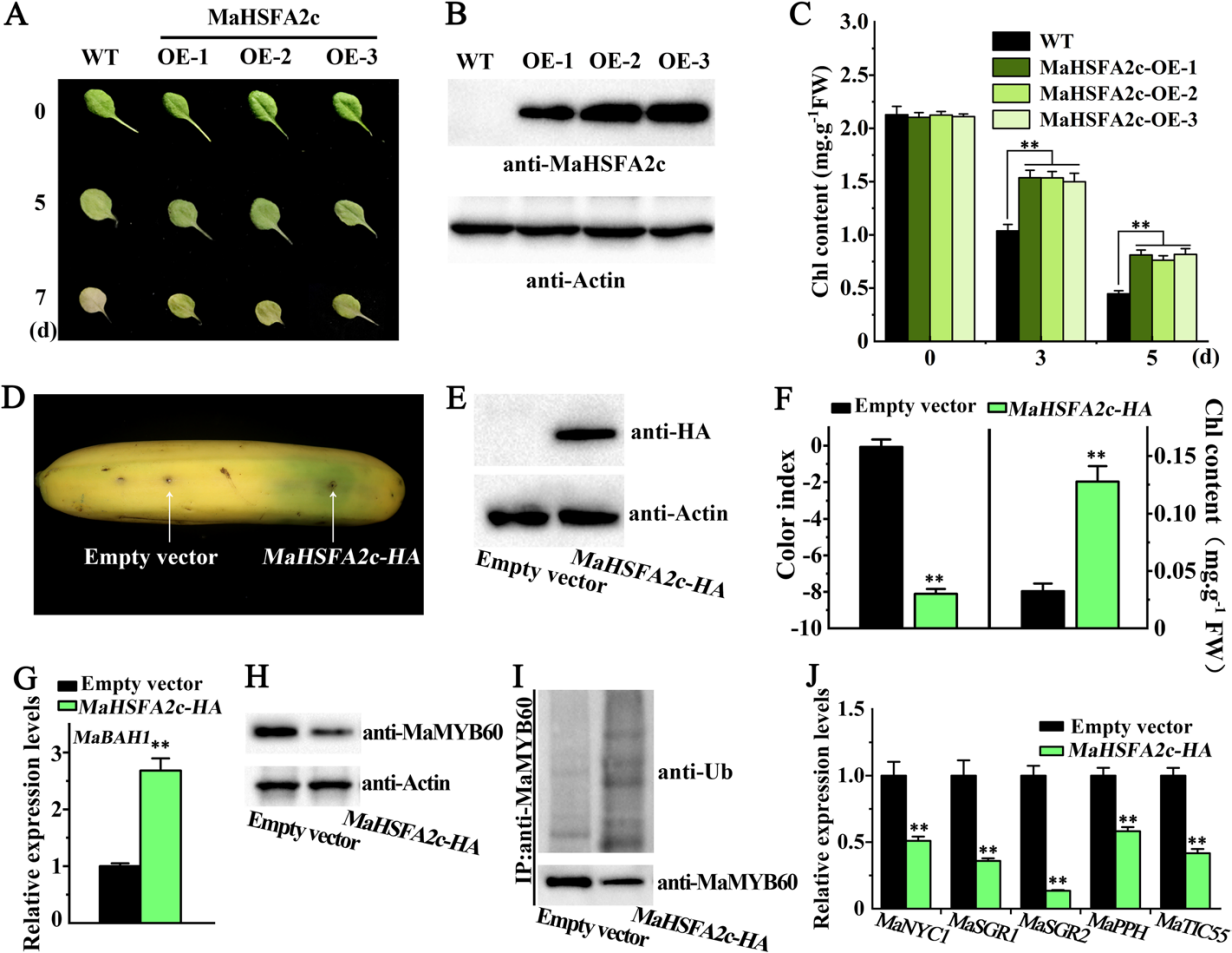
*MaHSFA2c* overexpression inhibits chlorophyll degradation. **A**-**C** Overexpression of *MaHSFA2c* delays leaf yellowing in *Arabidopsis*. **A** Leaf color phenotypes of wild-type (WT) as well as *MaHSFA2c*-overexpressing lines (OE-1/2/3) during dark-induced senescence at 0, 5, and 7 days. **B** Immunoblot analysis of MaHSFA2c in overexpressing plants (antibody: anti-MaHSFA2c; loading control: Actin). **C** Total chlorophyll content in leaves of WT and *MaHSFA2c*-OE plants during senescence. **D**-**G** Transient *MaHSFA2c* overexpression in banana fruit induces *MaBAH1* expression and reduces chlorophyll degradation. **D** Banana fruit infiltrated with an empty vector (left) and MaHSFA2c-HA (right) five days post-ethylene treatment. **E** Western blotting: MaHSFA2c-HA in banana peels. **F** Changes in peel color index and total chlorophyll content in bananas illustrated in (**D**). **G** *MaBAH1* expression in banana peels illustrated in (**D**). **H** Accumulation of endogenous MaMYB60 protein in banana peels illustrated in (**D**). **I** The ubiquitination level of MaMYB60 in banana peels illustrated in (**D**). **J** Expression of MaMYB60 target genes, including *MaNYC1*, *MaSGR1*/*2*, *MaPPH*, and *MaTIC55*, in banana peels illustrated in (**D**). Error bars represent SE (*n* = 6 for (**C**) and (**F**); *n* = 3 for (**G**) and (**J**)). Asterisks: Significance (** *p* < 0.01, Student's *t*-test)

To verify the potential involvement of MaHSFA2c in chlorophyll degradation, we generated MaHSFA2c overexpressing lines in an *Arabidopsis* WT background. At least three genetically independent homozygous transgenic lines were selected and verified by Western blot analysis (Figs. 4A-B). Transgenic lines overexpressing *MaHSFA2c* demonstrated increased leaf longevity and delayed yellowing compared to WT plants (Fig. 4A). Consistent with the observed phenotypes, *MaHSFA2c* overexpressing leaves exhibited significantly higher total chlorophyll content compared to WT leaves (Fig. 4C). These results suggest that MaHSFA2c negatively regulates chlorophyll degradation.

Since MaHSFA2c showed transcriptional activation activity and directly bound to the *MaBAH1* promoter (Figs. 1 and 3), *MaHSFA2c* was transiently overexpressed in banana peels to evaluate its role in activating *MaBAH1* expression and regulating banana fruit de-greening (Figs. 4D-E). Western blotting confirmed successful overexpression of MaHSFA2c-HA (Fig. 4E). Following ethylene treatment, transient overexpression of *MaHSFA2c-HA* in banana peel resulted in a stay-green phenotype at the injection site, in contrast to the yellow peel observed at the site inoculated with the empty vector (Fig. 4D). The side injected with MaHSFA2c exhibited a reduced color index and increased chlorophyll content compared to the side injected with the empty vector (Fig. 4F). Additionally, the transcripts of *MaBAH1* up-regulated in the peel transiently overexpressing *MaHSFA2c* (Fig. 4G). Immunoblotting analysis revealed that *MaHSFA2c* overexpression notably reduced MaMYB60 protein levels, the substrate of MaBAH1, in banana peel (Fig. 4H). We further tested the ubiquitination levels of MaMYB60 in peel transiently over-expressing *MaHSFA2c*. As shown in Fig. 4I, after immunoprecipitation of anti-MaMYB60, we used an anti-Ub antibody to detect poly-ubiquitinated MaMYB60, which was present in significantly higher quantities in the MaHSFA2c injected side compared to the side injected with the empty vector, indicating that overexpression of *MaHSFA2c* promotes MaMYB60 ubiquitination. Moreover, the overexpression of *MaHSFA2c* led to a significant decrease in the expression levels of MaMYB60's target genes, including chlorophyll catabolic gene (CCG) *MaNYC1* (*NON-YELLOW COLORING 1*), *MaPPH* (*PHEOPHYTINASE*), *MaTIC55* (*TRANSLOCON AT THE INNER ENVELOPE MEMBRANE OF CHLOROPLASTS 55*), *MaSGR1* (*STAY-GREEN 1*) and *MaSGR2* (*STAY-GREEN 2*) (Fig. 4J). Collectively, these findings reveal that MaHSFA2c induced the expression of *MaBAH1*, which in turn degraded MaMYB60 through ubiquitination and thereby inhibited CCGs' expression and chlorophyll degradation.

### Silencing of *MaHSFA2c* weakens high-temperature-induced green ripening

**Fig. 5:**
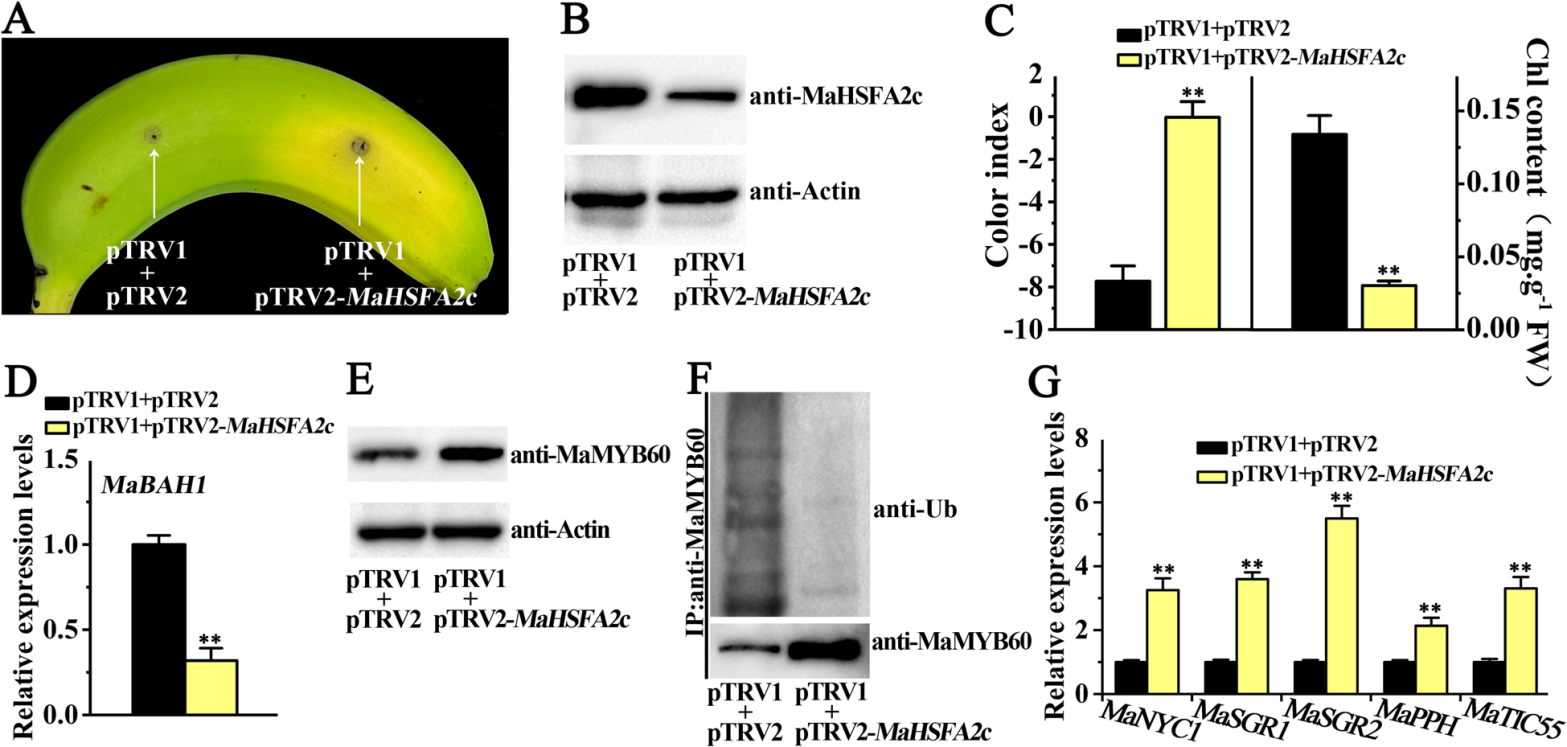
Transient VIGS of *MaHSFA2c* in banana fruit reduces high-temperature-induced green ripening. **A** The visual phenotype of banana fruit infiltrated with an empty vector (left) or pTRV2-MaHSFA2c (right) after 5 days at 30 °C. **B** Western blotting: MaHSFA2c protein in infiltrated banana peels (loading control: Actin). **C** Changes in the color index and total chlorophyll content in banana peels illustrated in (**A**). **D** *MaBAH1* expression levels in banana peels illustrated in (**A**). **E** Endogenous MaMYB60 protein accumulation in banana peels illustrated in (**A**). **F** The ubiquitination level of MaMYB60 in banana peels illustrated in (**A**). **G** Expression of MaMYB60 target genes, including *MaNYC1*, *MaSGR1/2*, *MaPPH*, and *MaTIC55*, in banana peels illustrated in (**A**). Error bars represent SE (*n* = 6 for (**C**); *n* = 3 for (**D**) and(**G**)). Asterisks: Significance (**p < 0.01, Student's *t*-test)

Herein, to study its role in green ripening induced by high temperature, we utilized VIGS to silence *MaHSFA2c* expression in banana peels and then subjected the fruits to 30℃ temperature treatment. (Figs. 5A–B). Unlike the empty vector, silencing *MaHSFA2c* in banana peels caused a significant de-greening phenotype near the injection site when ripened at 30 °C (Fig. 5A). Concurrently, the CI was observed to be lower. At the same time, the total chlorophyll content was higher in the *MaHSFA2c*-silenced area in comparison to the area injected with the empty vector following 30 °C treatment (Fig. 5C). In addition, the transcripts of *MaBAH1* were significantly downregulated in peel silencing *MaHSFA2c* at 30 °C (Fig. 5D). Further, silencing of *MaHSFA2c* significantly up-regulated the protein levels of MaMB60 when the fruit ripened at 30 °C (Fig. 5E). Simultaneously, the ubiquitination levels of MaMYB60 were significantly downregulated in peel silencing *MaHSFA2c* (Fig. 5F). Furtherly, the expression of CCGs, including *MaNYC1*, *MaSGR1/2*, *MaPPH*, and *MaTIC55*, which are downstream targets of MaMYB60, were significantly increased in the *MaHSFA2c*-silenced sections of the peel under HT (Fig. 5G). Thus, the findings demonstrate that MaHSFA2c positively regulates the repression of chlorophyll catabolism induced by HT through the upregulation of *MaBAH1* expression, which in turn inhibits the MaMYB60-CCGs module via promoting MaMYB60 ubiquitination and degradation.

## Discussion

Heat stress is one of the most concerning problems in the world today due to global warming and is a severe environmental factor contributing to fruit quality deterioration (Rezaei et al. 2021). Bananas are widely grown in tropical areas, but their fruit is extremely sensitive to heat stress (Yang et al. 2009). Ripening bananas at temperatures exceeding 24 °C accelerates fruit softening while inhibiting chlorophyll breakdown, resulting in a green peel and a green ripening phenotype (Luo et al. 2023). Our study reveals that elevated temperatures trigger the expression of the E3 ligase gene *MaBAH1*, facilitating the degradation of MaMYB60. This process suppresses MaMYB60's activation of chlorophyll catabolic genes, ultimately inhibiting peel de-greening in ripening bananas (Wei et al. 2023a, b, c). There is currently a lack of understanding regarding the mechanism underling the induction of *MaBAH1* by heat stress. Here, we identified MaHSFA2c as an upstream transcription regulator of *MaBAH1* and discussed the regulatory function of the MaHSFA2c-MaBAH1 module in HT-caused green ripening of banana fruit.

Plant resistance to extreme temperatures is an intricate process governed by diverse transcription factors that involve the selective expression of numerous stress-protective genes (Ding et al. 2020). HSFs, as primary transcription factors functioning upstream of temperature stress signal transduction pathways, regulate the transcription of stress-protective genes that help protect against stress (Andrási et al. 2021) and are involved in responses to heat stress in several fruits, such as citrus (Lin et al. 2015), tomato (Rao et al. 2022) and apple (Zhang et al. 2024). Notably, HSFs have also been found to regulate changes in fruit quality under extreme temperature stresses. For example, loquat EjHSF1 modulates lignin biosynthesis under chilling stress by directly inhibiting *EjPAL1* and *Ej4CL1/5* expression (Zeng et al. 2016). Additionally, citrus CitHsfA7/A3/A4b/A8 regulate citrate degradation in fruit under heat stress by directly inducing the expression of *CitAco3*, *CitIDH3*, and *CitGAD4* (Li et al. 2022). Recent studies have identified that banana MaHsf24/26 transcriptionally regulates antioxidant and respiratory burst oxidase homolog genes, respectively, contributing to the mitigation of chilling injury in banana fruit under heat shock conditions (Si et al. 2022; 2024). Nonetheless, the implications of HSFs in fruit quality deterioration due to extreme temperatures remain unclear. The present study demonstrated that MaHSFA2c gene expression and protein accumulation were induced at 30 °C, correlating with the green ripening phenotype under HT (Fig. 2). In addition, *MaHSFA2c* was most highly expressed in banana fruit at 30 °C among different tissues, including root, stem, leaf and fruit at both 20 and 30 °C (Fig. S4), suggesting that MaHSFA2c exerts its action mainly in fruit under HT. Moreover, stable *MaHSFA2c*-overexpressing *Arabidopsis* exhibited a delayed leaf yellowing phenotype and increased chlorophyll content. In addition, transient overexpression of *MaHSFA2c* in banana fruit inhibited chlorophyll degradation and resulted in a stay-green phenotype after the fruit ripened following ethylene treatment (Fig. 4). However, the suppression of chlorophyll degradation at HT was diminished when *MaHSFA2c* was silenced in banana peels, leading to a significant de-greening phenotype during the ripening process at a temperature of 30 °C (Fig. 5). Specifically, these findings suggest that MaHSFA2c functions as a negative regulator in the chlorophyll degradation pathway, influencing the de-greening of fruit and contributing to the green-ripening process induced by HT in bananas. This discovery enhances our understanding of the regulatory mechanisms in fruit deterioration due to heat stress and expands the HSF regulatory network.

Protein ubiquitination is a crucial post-translational mechanism that regulates selective protein degradation in eukaryotic cells (Xu And Xue 2019). E3 ubiquitin ligases play a vital role in ubiquitination and significantly influence fruit ripening and quality-related metabolic processes, including pigment biosynthesis and degradation (Muhammad et al. 2024). PPSR1, a RING finger E3-ubiquitin ligase, ubiquitinates and degrades the carotenoid biosynthetic enzyme PSY1, thereby negatively regulating carotenoid accumulation in tomato fruit (Wang et al. 2020). MdPUB24, an apple U-box type E3 ligase, enhances chlorophyll degradation in fruit by facilitating the breakdown of MdBEL7, a transcriptional repressor of chlorophyll catabolic genes (Wei et al. 2021). MdMIEL1 is responsible for the degradation of MdMYB308L, which consequently has a detrimental impact on cold tolerance as well as anthocyanin accumulation in apple fruit (An et al. 2020). Wei et al. (2023a) discovered that elevated temperatures trigger MaBAH1-mediated degradation of MaMYB60, which suppresses MdMYB60-driven transactivation of CCGs and chlorophyll degradation in bananas. Although several E3s have been identified as key regulators of fruit coloration during ripening and fruit color deterioration under stress conditions, the molecular mechanisms of how these E3s respond to ripening and stress are not completely understood, especially regarding their upstream regulators. In our study, a heat stress-induced MaHSFA2c was found to target the *MaBAH1* promoter (Figs. 1 and 2). MaHSFA2c possessed transcriptional activation activity (Fig. 3) and induced the expression of *MaBAH1* to down-regulate the MaMYB60 protein and its downstream CCGs (Fig. 4). Crucially, silencing *MaHSFA2c* in banana fruit inhibited MaBAH1's role in degrading MaMYB60, thereby enhancing MaMYB60's induction of chlorophyll degradation and mitigating the green-ripening symptoms caused by HT (Fig. 5). Taken together, our data establish that HT induces MaHSFA2c to activate the MaBAH1-MaMYB60-CCGs module, thereby repressing chlorophyll degradation, and unraveling a new upstream regulatory mechanism controlling fruit color deterioration under heat stress.

It is worth pointing out that different HSFs can construct complicated transcriptional cascades to transduce stress signals (Shekhawat et al. 2022). MaHSFA2c belongs to class A HSFs (Fig. S1), which have been reported to be regulated by class B HSF members (Ikeda et al. 2011; Zhao et al. 2022). Recently, the banana HSFB members MaHsf24 and MaHsf26 have been identified as upstream regulators in the cold response of fruit (Si et al. 2022; 2024). Thus, it will be an interesting aspect of future research to investigate whether these HSFB members or other banana class B HSFs can regulate MaHSFA2c during banana green-ripening under heat stress.

**Fig. 6:**
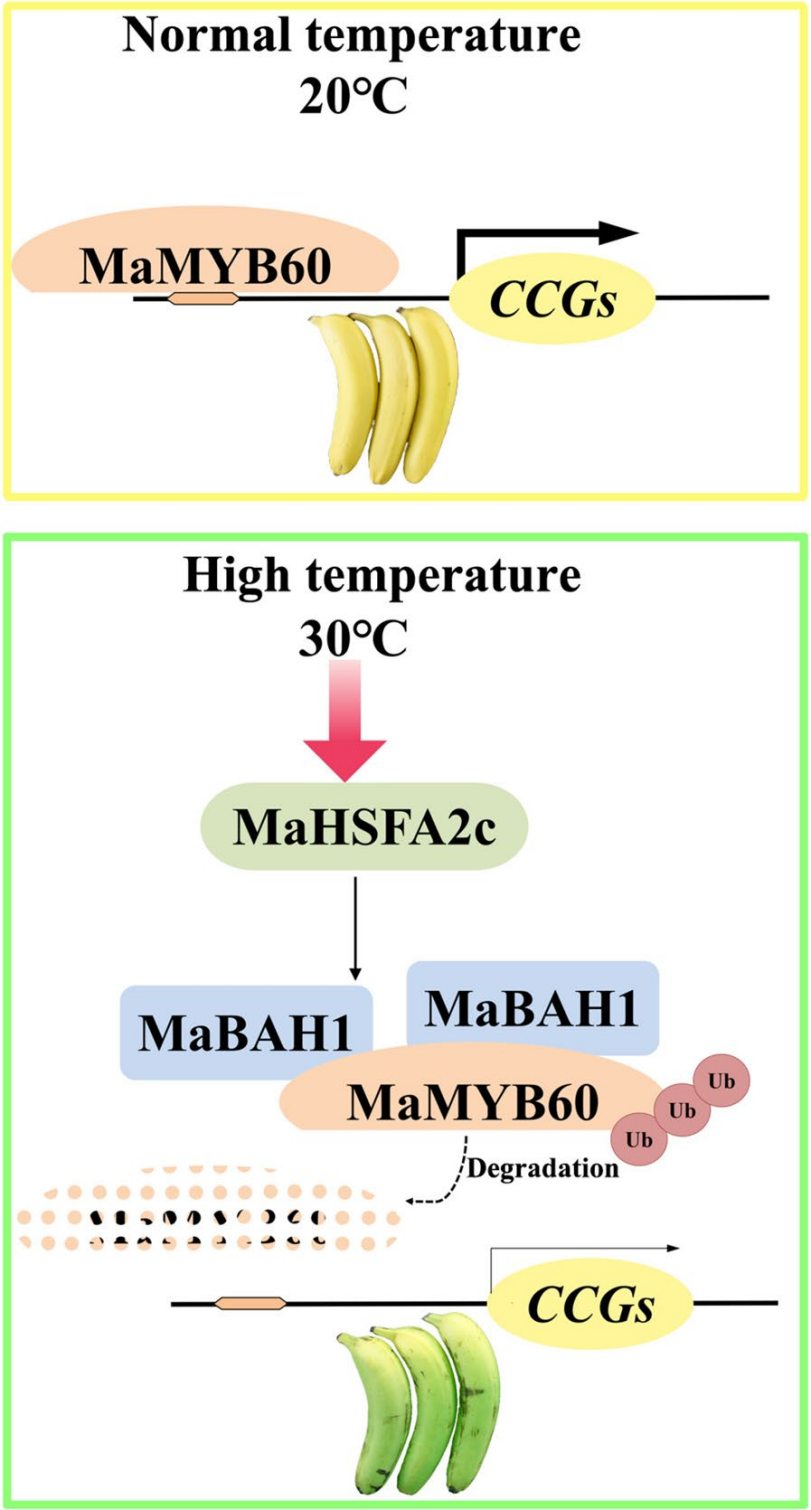
Proposed model for the MaHSFA2c-MaBAH1 module regulating high-temperature-provoked green ripening in banana fruit. When the banana ripen at normal temperatures (e.g., 20 °C), MaMYB60 induces the expression of CCGs to promote chlorophyll degradation, leading to de-greening and a yellow peel. When banana ripen at high temperatures (e.g., 30 °C), MaHSFA2c is activated. MaHSFA2c directly activates the transcription of MaBAH1 to promote MaBAH1-mediated degradation of MaMYB60 and then blocks CCGs’ expression. As a result, chlorophyll degradation is inhibited and banana fruit fails to develop a yellow peel and green ripens

Building on current and prior research, we suggest a model illustrating how MaHSFA2c influences the green-ripening process in bananas (Fig. 6). At normal ripening temperatures (e.g. 20 °C), MaMYB60 triggers the expression of CCGs, facilitating chlorophyll breakdown and resulting in the fruit's de-greening and yellow peel appearance. MaHSFA2c is activated when bananas ripen at elevated temperatures, such as 30 °C. It activates *MaBAH*1 transcription, leading to MaMYB60 degradation and subsequent inhibition of CCG expression. Consequently, chlorophyll degradation is inhibited, preventing banana fruit from developing a yellow peel and causing them to ripen while remaining green. Our findings uncover a novel regulatory module, MaHSFA2c-MaBAH1, which controls chlorophyll degradation inhibited by HT in banana fruit. This enhances the understanding of transcriptional regulation's role in fruit quality deterioration due to temperature stress.

## Materials and methods

### Plant materials and growth conditions

The Cavendish banana variety Brazilian (*Musa* spp. AAA group) fruit was harvested from a commercial plantation located close to Guangzhou, China, and its pre-climacteric stage was collected. The ripening process began by administering 100 µL L^–1^ of ethylene, as well as the fruit was subsequently given the opportunity to mature at temperatures of either 20 °C or 30 °C for a duration of six days. The pre-ripening initiation fruit served as the day 0 samples. Peel color was measured by diffuse reflectance using a chroma meter. The chroma meter allocates color coordinates to each sample using the 3-dimensional L* × a* × b* color space, and the color index was further calculated by following the formula: 1000 × a*/L* × b*. At each sampling time, we recorded color index and chlorophyll content using methods previously described by Wei et al. (2024).

Wild-type (WT) and transgenic *Arabidopsis thaliana* (ecotype Columbia) were cultivated in soil within a growth chamber at 21 °C, under cool-white fluorescent lighting, following a 16:8-h light: dark cycle.

### Gene expression and western blot analysis

Total RNA extraction from banana fruits was performed using the RNeasy Plant Kit (Qiagen, Germany) while cDNA was synthesized via reverse transcription with the TaKaRa PrimeScript reagent kit. qRT- PCR was conducted on a Bio-Rad CFX96 system with the SYBR Green PCR Supermix Kit (Bio-Rad) according to the established protocols. The transcript levels of each gene were normalized using *MaACT1* (Chen et al. 2011) for bananas.

To produce anti-MaHSFA2c and anti-MaMYB60 antibodies, recombinant His-tagged MaHSFA2c and MaMYB60 were produced in *Escherichia coli* strain BM Rosetta, purified using Ni-NTA agarose (GE Healthcare) and separated on SDS-PAGE gel. Bands of interest were excised and used as antigens to immunize rabbits. Each polyclonal antibody was affinity-purified from rabbit antisera by Hangzhou HuaAn Biotechnology Co., Ltd (Hangzhou, China). To extract protein, a kit from Bangfei, China was used. The specificities of the anti-MaHSFA2c and anti-MaMYB60 antibodies were validated by immunoblotting analysis using total protein extracts from banana fruit peel, in vitro-translated recombinant protein, and total protein extracts from tobacco leaves overexpressing *MaHSFA2c* or *MaMYB60* (Figs. S5-S6). Protein separation was conducted via SDS-PAGE with 30 mg of protein loaded per lane. Following electrophoresis, the protein was transferred onto the nitrocellulose membrane (Thermo). Aiming at conducting western blotting, we employed an anti-MaHSFA2c antibody and a goat anti-rabbit secondary antibody (Thermo Scientific).

### Y1H

The Y1H screening utilized the Matchmaker™ Gold Yeast One-Hybrid System (Clontech). The pAbAi vector was used to create the bait plasmid by inserting the *MaBAH1* promoter fragment. Following linearization, the construct was injected into Y1H Gold yeast, resulting in the production of a reporter strain specific to the bait. This was followed by screening a cDNA library from banana peels. DNA–protein interactions were evaluated by examining the maturation potential of co-transformants on SD/-Leu medium supplemented with Aureobasidin A (AbA) per established protocols.

### EMSA

MaHSFA2c with a glutathione S-transferase (GST) tag was produced in *Escherichia coli* BM Rosetta (DE3) and subsequently purified using glutathione-Sepharose 4B beads. The segment containing heat shock elements (HSEs) in the promoter of *MaBAH1* was generated and biotinylated at the 5' terminus. EMSA was conducted using a Chemiluminescent EMSA kit (Thermo), as described in Wei et al. (2023b). Biotin-labeled probes were incubated with the GST-MaHSFA2c recombinant protein in a binding buffer, followed by the separation of unbound and bound probes using an acrylamide gel. Unlabeled probes served as competitors, while the GST protein acted as a negative control.

### ChIP-qPCR analysis

For the purpose of cross-linking genomic proteins and their DNA, the peel of the banana fruit was suspended in 1% formaldehyde. Chromatin was sonicated to achieve a length of approximately 500 bp. The DNA–protein complex was then immunoprecipitated using an anti-MaHSFA2c polyclonal antibody, while a nonspecific IgG antibody was used as a negative control.

### Transient expression analysis in tobacco leaf

The subcellular localization and transcriptional capability assays were conducted in tobacco leaf cells, as mentioned earlier (Wei et al. 2023a). The coding regions of MaHSFA2c were cloned into the pEAQ-GFP vector to create a fusion with the GFP reporter gene for subcellular localization assays. The fusion protein plasmids, the nuclear marker nucleus-targeted mCherry (NLS-mCherry), and the control pEAQ-GFP vector were electroporated into *Agrobacterium tumefaciens* strain EHA105. Agrobacteria were cultured for 12 to 16 h and then injected into tobacco using a 1 mL syringe. After 36 h of infiltration, we imaged the GFP and mCherry fluorescent signals with a Zeiss fluorescence microscope.

To evaluate the transcriptional capability, the full-length coding sequence of the MaHSFa2c was loned into the pGreenII 62SK-BD vector was performed. This double-reporter vector includes a firefly luciferase (LUC) gene regulated by five GAL4-binding elements. The effector and reporter were co-transferred into tobacco leaves, as previously outlined. Both LUC and REN activities were quantified 60 h after injection.

### Transcriptional activation assay in yeast

The MaHSFA2c coding sequences were cloned into the pGBKT7 vector. The fusion plasmid, along with the positive control (pGBKT7-53 + pGADT7-T) and the negative control (pGBKT7 vector), was independently transformed into the Y2H yeast using the lithium acetate method. The growth status and X-α-galactosidase activity in the SD medium lacking tryptophan, histidine, and adenine indicated the transcriptional activity of MaHSFA2c.

### Stable overexpression analysis in Arabidopsis

To overexpress *MaHSFA2c* in Arabidopsis, we inserted the full-length cDNA for MaHSFA2c into the pCAMBIA1302 vector, and the resulting vector was introduced into Col plants via the floral-dip method. Transgenic plants with elevated *MaHSFA2c* expression were confirmed through analysis of MaHSFA2c protein levels. In the context of this research, three distinct homozygous lines (T3) were utilized. Following the procedure described by Sakuraba et al. (2014), detached rosette leaves from three week-old plants were placed in completely darkness in an incubator. Photographic analysis and chlorophyll measurements were conducted on days 0, 5, and 7 of dark incubation.

### Transient overexpression analysis in banana peel

The cloning of the *MaHSFA2c* open reading frame into the pCXUN-HA vector was followed by being transferred into *Agrobacterium tumefaciens* EHA105 and injected into mature green banana fruit peel. At least 30 fruits were injected with the transformed *Agrobacterium* and the transient expression assays were repeated six times. One day post-inoculation, the transformed fruits were exposed to 100 µL L^–1^ ethylene and maintained at 20 °C for 5 days. The peel surrounding the injection sites was utilized to measure color index, chlorophyll content, gene expression, and protein accumulation. The protein extracts from the injection sites were immunoprecipitated with anti-MaMYB60 and subjected to immunoblotting using an anti-ubiquitin antibody for the ubiquitination assay.

### Virus-induced gene silencing (VIGS) in banana peel

The cDNA fragment of *MaHSFA2c* was incorporated directly into the pTRV2 vector. The peels of mature green banana fruits were injected with a mixture of *Agrobacterium tumefaciens* EHA105 containing pTRV2-MaHSFA2c and pTRV1, as described in Wei et al. (2023c). The inoculated fruits were treated with ethylene and maintained at 30 °C for 5 days. Samples were taken from the peel around the injection sites and analyzed as detailed above.

### Statistical analysis

Statistical analysis was conducted through SPSS19.0, reporting data as mean ± standard errors (SE) of three or six independent replicates. Statistical differences between samples were ascertained using Student's *t*-test, with *P* < 0.05 or 0.01 indicating significance.

### Primes

Table S1 lists the used primers.

## Supplementary Information

Supplementary Material 1: Figure S1. Phylogenetic and sequence analyses of MaHSFA2c. (A) A phylogenetic analysis of HSFs from Musa acuminata (banana), Oryza sativa (rice) and Arabidopsis thaliana (Arabidopsis). MaHSFA2c from banana was indicated by black dot. The phylogenetic tree was constructed using a bootstrap test of phylogeny with Neighbor-Joining test in MEGA6.0. Gene code of proteins used for this analysis are listed in Table S2. (B) Protein sequence analyses of MaHSFA2c with rice and Arabidopsis HSFs proteins, including AtHSFA2 (AT2G26150), OsHSFA2a (LOC_Os03g53340), OsHSFA2b (LOC_Os07g08140), OsHSFA2c (LOC_Os10g28340) and OsHSFA2e (LOC_Os03g58160). Identical and similar amino acids are shaded in black and gray, respectively. The conserved DNA binding domain (DBD, α1-β1-β2-α2-α3-β3-β4) are shown above. The heptad hydrophobic repeat (HR-A/B) motif and nuclear localization signal (NLS) are highlighted by black bold lines. Figure S2. HSE in the MaBAH1 promoter. HSEs are indicated by diamonds. The probe nucleotide sequence used in EMSA are listed below and the containing HSE are identified in red font. The numbers indicate the start and end positions of EMSA probes. Figure S3. The prokaryon-expressed and purified GST-HSFA2c recombinant protein was visualized on a Coomassie blue-stained SDS-PAGE. Lane 1: non-induced protein; Lane 2: before purification of the induced protein; Lane 3: after purification of the induced protein. Figure S4. Changes of MaHSFA2c ranscription levels in different tissues at 20 and 30 °C. The relative mRNA abundance in different tissues was expressed as a ratio relative to that of the root at 20 °C, which was set at 1. Error bars represent SE (*n* = 3). Asterisks: Significance (** *p* < 0.01, Student's t-test). Figure S5. Western blotting analysis of anti-MaHSFA2c antibody specificity. Total protein from banana fruit (lane 1), in vitro-translated recombinant protein of GST-MaHSFA2c (lane 2) and total protein from MaHSFA2c-GFP-overexpressing tobacco leaves (lane 3) were hybridized with anti-MaHSFA2c polyclonal antibody. GST (lane 5) was used as the negative control. Figure S6. Western blotting analysis of anti-MaMYB60 antibody specificity. Total protein from banana fruit (lane 1), in vitro-translated recombinant protein of GST-MaMYB60 (lane 2) and total protein from MaMYB60-GFP-overexpressing tobacco leaves (lane 3) were hybridized with anti-MaMYB60 polyclonal antibody. GST (lane 5) was used as the negative control. Table S1. Summary of primers used in this study. Table S2. Gene codes of HSFs used for phylogenetic analysis.

## Data Availability

The datasets used and/or analyzed during the present study are available from the corresponding author on reasonable request.

## Abbreviations

HT: High temperature
Y1H: Yeast one-hybrid
Y2H: Yeast two-hybrid
HSE: Heat shock element
EMSA: Electrophoretic mobility shift assay
ChIP: Chromatin immunoprecipitation
PCR: Polymerase chain reaction
CI: Color index
NLS: Nuclear localization signal
GFP: Green fluorescent protein
CCG: Chlorophyll catabolic gene
VIGS: Virus-induced gene silencing
WT: Wild type
GST: Glutathione S-transferase
SDS-PAGE: Sodium dodecyl sulfate polyacrylamide gel electrophoresis
LUC: Firefly luciferase
REN: Renilla luciferase
