# Supplementary material for: MaHSFA2c modulates high temperature-inhibited chlorophyll breakdown in banana fruit by directly inducing the transcription of the E3 ligase gene MaBAH1
Source: Mol Hortic. 2026 Apr 3;6:26. doi: 10.1186/s43897-025-00209-4 (PMC13047763; doi:10.1186/s43897-025-00209-4)
Supplement: Supplementary file 1 — Supplementary Material 1: Figure S1. Phylogenetic and sequence analyses of MaHSFA2c. (A) A phylogenetic analysis of HSFs from Musa acuminata (banana), Oryza sativa (rice) and Arabidopsis thaliana (Arabidopsis). MaHSFA2c from banana was indicated by black dot. The phylogenetic tree was constructed using a bootstrap test of phylogeny with Neighbor-Joining test in MEGA6.0. Gene code of proteins used for this analysis are listed in Table S2. (B) Protein sequence analyses of MaHSFA2c with rice and Arabidopsis HSFs proteins, including AtHSFA2 (AT2G26150), OsHSFA2a (LOC_Os03g53340), OsHSFA2b (LOC_Os07g08140), OsHSFA2c (LOC_Os10g28340) and OsHSFA2e (LOC_Os03g58160). Identical and similar amino acids are shaded in black and gray, respectively. The conserved DNA binding domain (DBD, α1-β1-β2-α2-α3-β3-β4) are shown above. The heptad hydrophobic repeat (HR-A/B) motif and nuclear localization signal (NLS) are highlighted by black bold lines. Figure S2. HSE in the MaBAH1 promoter. HSEs are indicated by diamonds. The probe nucleotide sequence used in EMSA are listed below and the containing HSE are identified in red font. The numbers indicate the start and end positions of EMSA probes. Figure S3. The prokaryon-expressed and purified GST-HSFA2c recombinant protein was visualized on a Coomassie blue-stained SDS-PAGE. Lane 1: non-induced protein; Lane 2: before purification of the induced protein; Lane 3: after purification of the induced protein. Figure S4. Changes of MaHSFA2c ranscription levels in different tissues at 20 and 30 °C. The relative mRNA abundance in different tissues was expressed as a ratio relative to that of the root at 20 °C, which was set at 1. Error bars represent SE (n = 3). Asterisks: Significance (** p < 0.01, Student's t-test). Figure S5. Western blotting analysis of anti-MaHSFA2c antibody specificity. Total protein from banana fruit (lane 1), in vitro-translated recombinant protein of GST-MaHSFA2c (lane 2) and total protein from MaHSFA2c-GFP-overexpressing [file 43897_2025_209_MOESM1_ESM.doc]

**Supplementary Material**


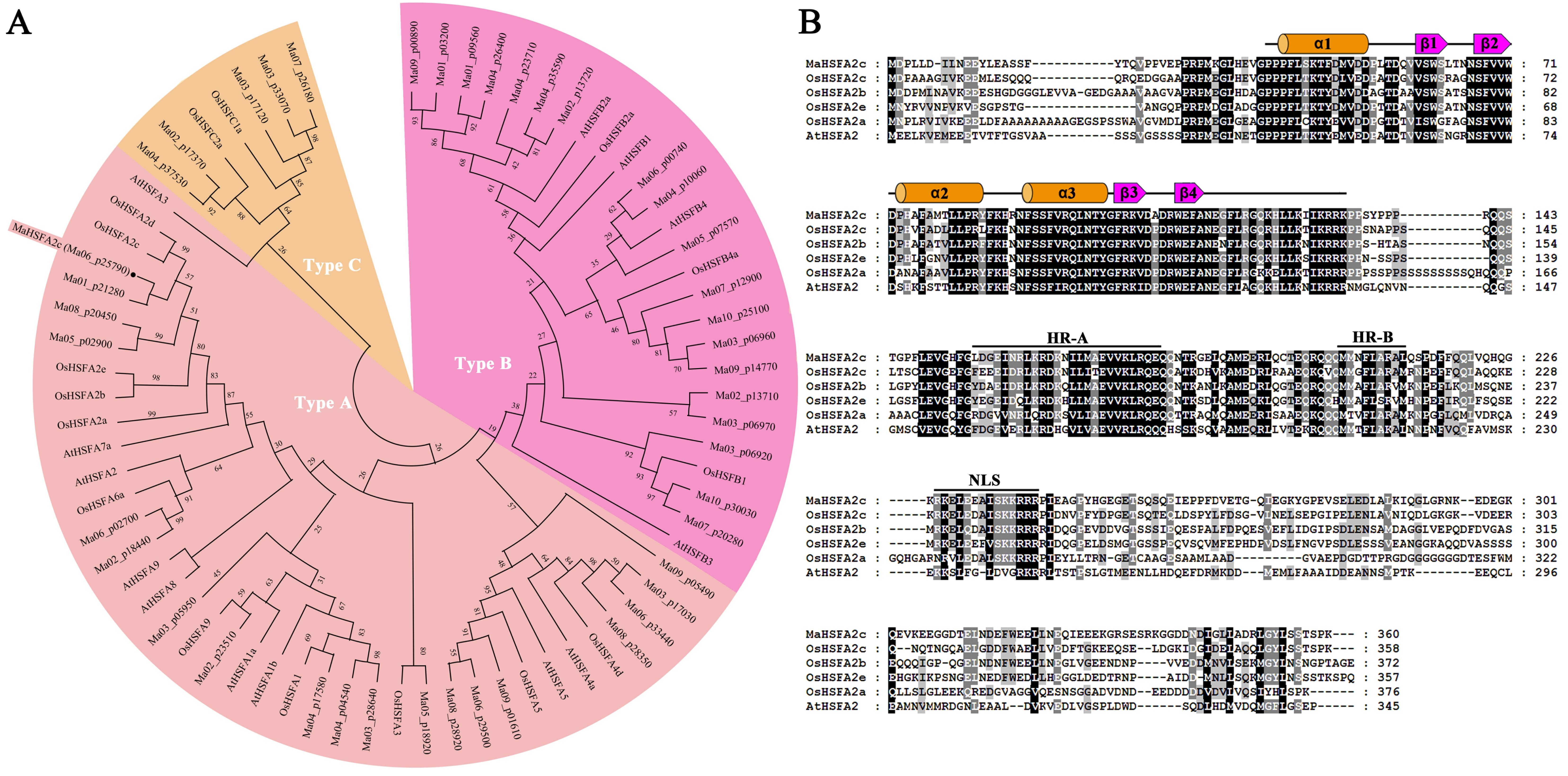


**Fig. S1.** Phylogenetic and sequence analyses of MaHSFA2c. **(A)** A phylogenetic analysis of HSFs from *Musa acuminata* (banana), *Oryza sativa* (rice) and *Arabidopsis thaliana* (Arabidopsis). MaHSFA2c from banana was indicated by black dot. The phylogenetic tree was constructed using a bootstrap test of phylogeny with Neighbor-Joining test in MEGA6.0. Gene code of proteins used for this analysis are listed in Table S2. **(B)** Protein sequence analyses of MaHSFA2c with rice and Arabidopsis HSFs proteins, including AtHSFA2 (AT2G26150), OsHSFA2a (LOC_Os03g53340), OsHSFA2b (LOC_Os07g08140), OsHSFA2c (LOC_Os10g28340) and OsHSFA2e (LOC_Os03g58160). Identical and similar amino acids are shaded in black and gray, respectively. The conserved DNA binding domain (DBD, α1-β1-β2-α2-α3-β3-β4) are shown above. The heptad hydrophobic repeat (HR-A/B) motif and nuclear localization signal (NLS) are highlighted by black bold lines.


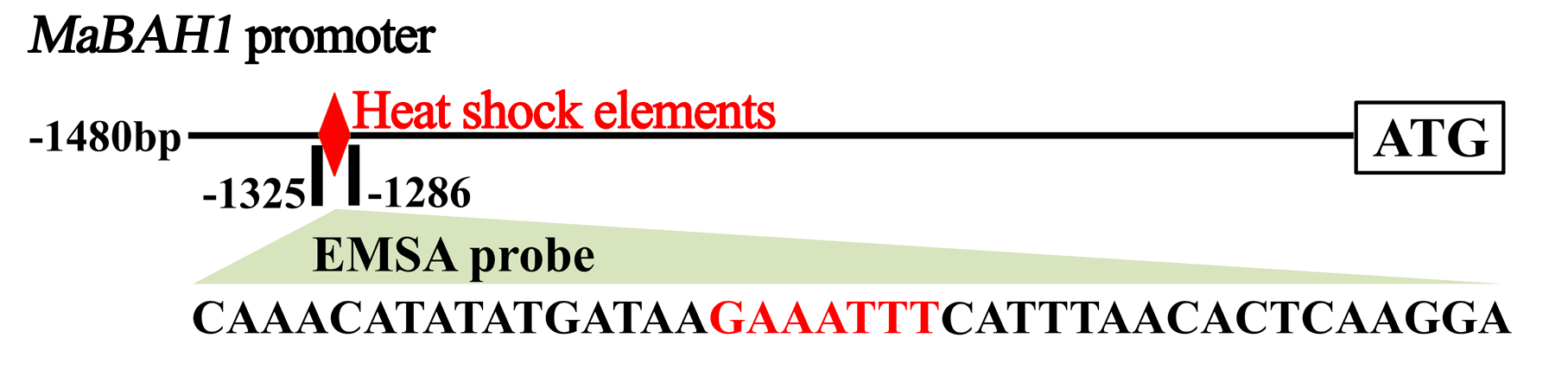


**Fig. S2.** HSE in the *MaBAH1* promoter. HSEs are indicated by diamonds. The probe nucleotide sequence used in EMSA are listed below and the containing HSE are identified in red font. The numbers indicate the start and end positions of EMSA probes.


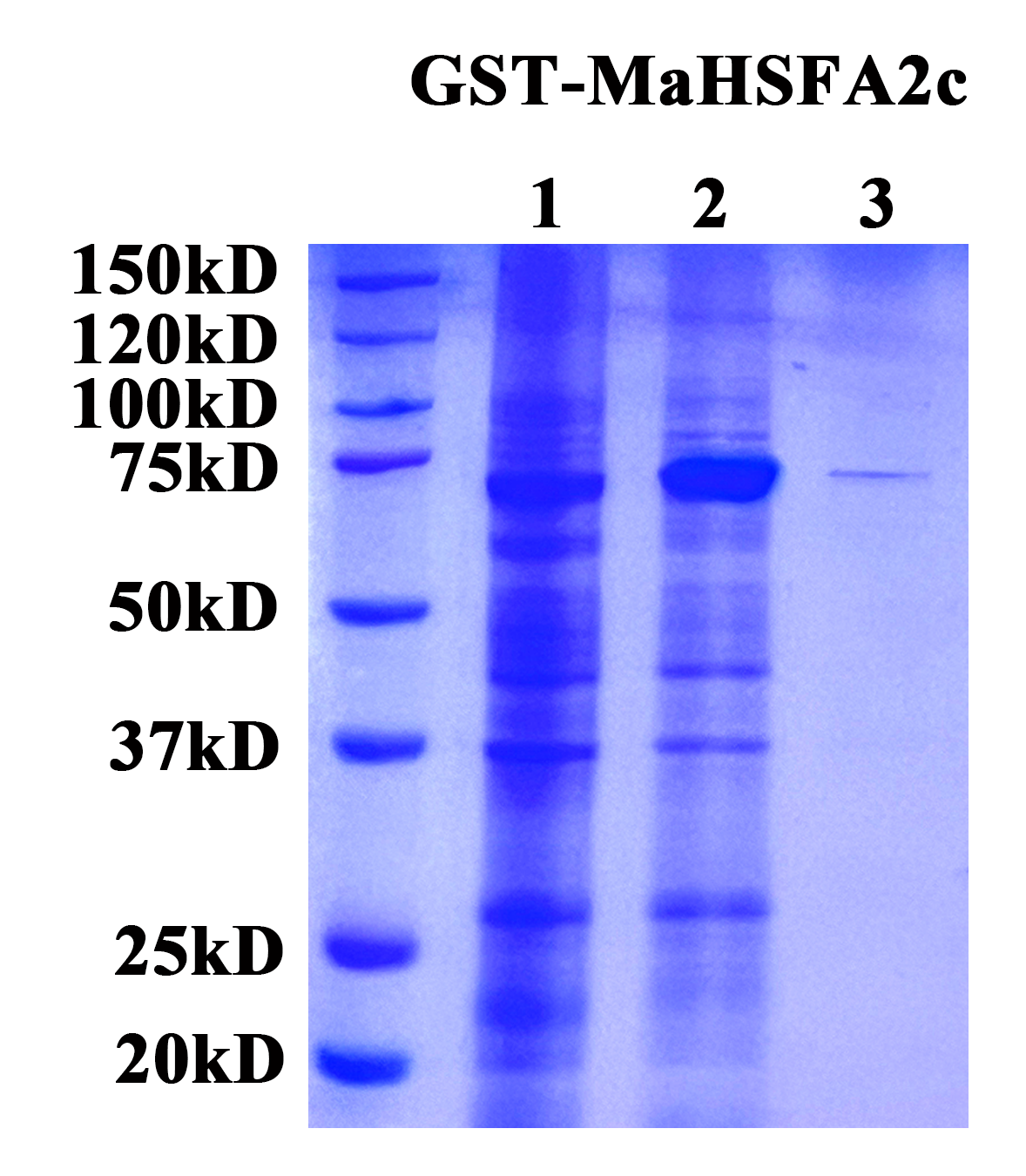


**Fig. S3.** The prokaryon-expressed and purified GST-HSFA2c recombinant protein was visualized on a Coomassie blue-stained SDS-PAGE. Lane 1: non-induced protein; Lane 2: before purification of the induced protein; Lane 3: after purification of the induced protein.


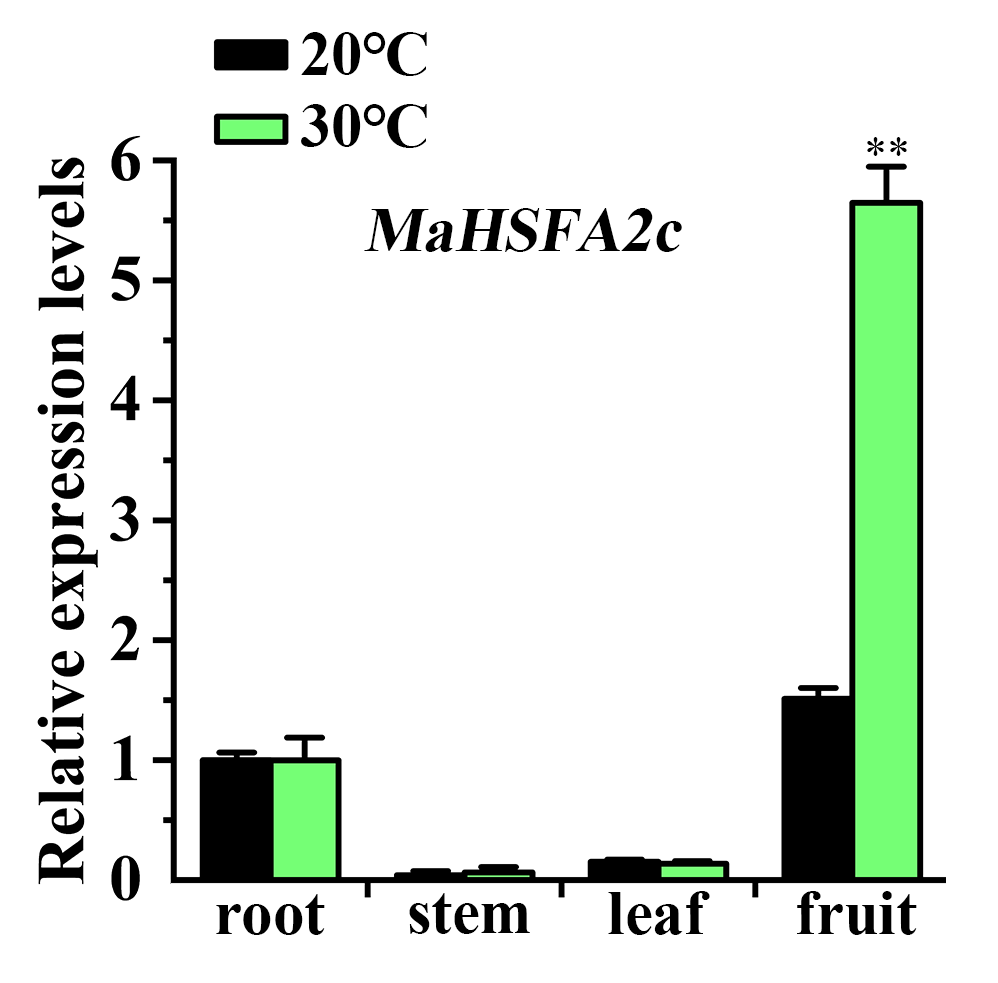


**Fig. S4.** Changes of *MaHSFA2c* ranscription levels in different tissues at 20 and 30 °C. The relative mRNA abundance in different tissues was expressed as a ratio relative to that of the root at 20°C, which was set at 1. Error bars represent SE (*n* =3). Asterisks: Significance (** *p* < 0.01, Student's *t*-test).


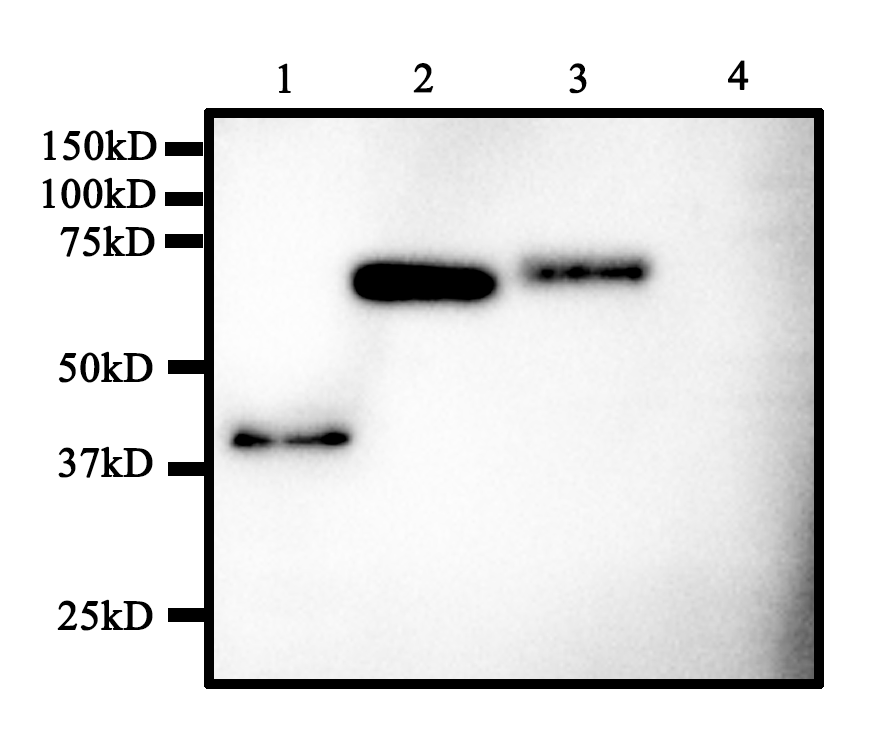


**Fig. S5.** Western blotting analysis of anti-MaHSFA2c antibody specificity. Total protein from banana fruit (lane 1), in vitro-translated recombinant protein of GST-MaHSFA2c (lane 2) and total protein from MaHSFA2c-GFP-overexpressing tobacco leaves (lane 3) were hybridized with anti-MaHSFA2c polyclonal antibody. GST (lane 5) was used as the negative control.


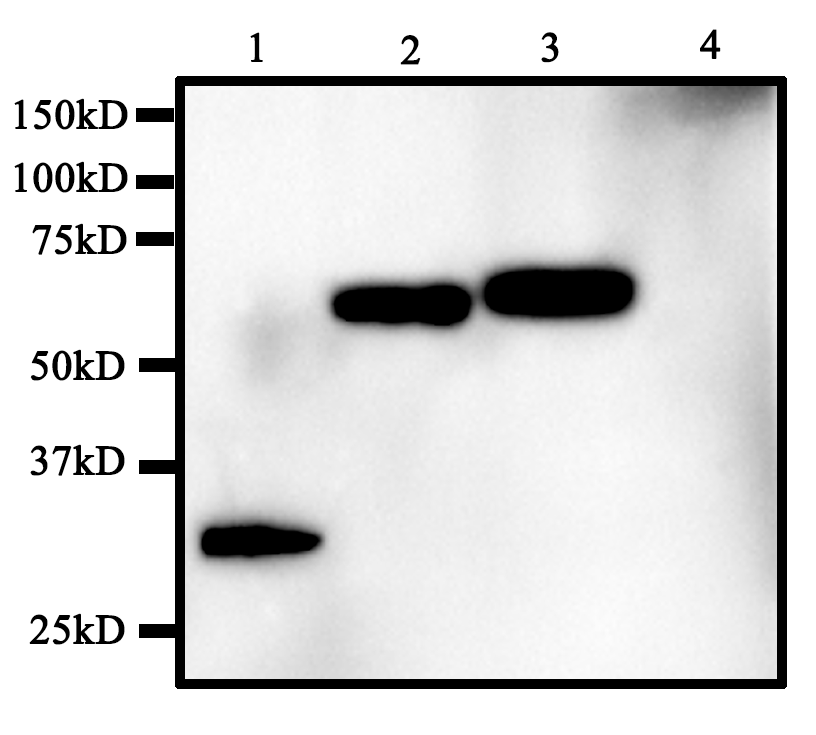


**Fig. S6.** Western blotting analysis of anti-MaMYB60 antibody specificity. Total protein from banana fruit (lane 1), in vitro-translated recombinant protein of GST-MaMYB60 (lane 2) and total protein from MaMYB60-GFP-overexpressing tobacco leaves (lane 3) were hybridized with anti-MaMYB60 polyclonal antibody. GST (lane 5) was used as the negative control.

**Table S1.** Summary of primers used in this study.

| **Assay** | **Primer sequence (5’-3’)** | **Restriction Site** |
| --- | --- | --- |
| **Full length cloning** | *MaHSFA2c-F:* ATGGATCCACTTCTCGATATACTGAACG  *MaHSFA2c-R:* CTACTTGGGACTAGTGGAACTAAGATAGCCT |  |
| **Subcellular localization** | *MaHSFA2c-GFP-F:* TATTCTGCCCAAATTCGCGACCGGTATGGATCCACTTCTCGATATACTGAACG  *MaHSFA2c-GFP-R:* AAAGTTCTTCTCCTTTGCTAGTCATCTTGGGACTAGTGGAACTAAGATAGCCT | *Age* I  *Age* I |
| **Yeast one-hybrid** | *MaBAH1-pAbAi-F*: AATTGAAAAGCTTGAATTCGAGCTCTCAAACATATATGATAAGAAATTTCAT  *MaBAH1-pAbAi -R*: ACAGAGCACATGCCTCGAGGTCGACAGTGAGAAGCCGGTGGG | *Sac* I  *Sal* I |
| **Yeast two-hybrid** | *MaHSFA2c-BD- F:* ATGGCCATGGAGGCCGAATTCATGGATCCACTTCTCGATATACTGAACG  *MaHSFA2c-BD- R:* TGCGGCCGCTGCAGGTCGACGCTTGGGACTAGTGGAACTAAGATAGCCT | *Eco*R I  *Sal* I |
| **RT-qPCR** | *MaACT1-qF:* TGGTATGGAAGCCGCTGGTA  *MaACT1-qR:* TCTGCTGGAATGTGCTGAGG  *MaHSFA2c-qF:* CGTGCTCTACAAAGCCCTGAC  *MaHSFA2c-qR:* CTGCCCTTCTCCTCTTCAATC |  |
| **Transient overexpression** | *MaHSFA2c-pCXUN-HA-F*: CATACGATGTTCCAGATTACGCTATGGATCCACTTCTCGATATACTGAACG  *MaHSFA2c-pCXUN-HA-R*: CATACGATGTTCCAGATTACGCTATGGATCCACTTCTCGATATACTGAACG  *MaHSFA2c-pTRV2-F*: GCCTCCATGGGGATCCGAGTTCCTTTTACACACAAGTTC  *MaHSFA2c-pTRV2-R*: CTTCGGGACATGCCCGGGTCATTTGCAAATTCCCATCT | *Xcm* I  *Xcm* I  *Bam*H I  *Sma* I |
| **Promoter isolation** | *MaBAH1 pro-F:* GGAGACTCCTACACCAAGAAATATGT  *MaBAH1 pro-R:* CATCTCGGATTTGAAGTGGG |  |
| **Dual-luciferase transient expression assay** | *MaHSFA2c-BD-62SK- F:* CGCCGTCTAGAACTAGTGGATCCATGGATCCACTTCTCGATATACTGAACG  *MaHSFA2c-BD-62SK- R:* TCGATAAGCTTGATATCGAATTCTCACTTGGGACTAGTGGAACTAAGATAGCCT | *Bam*H I  *Eco*R I |
| **EMSA assay** | *MaHSFA2c- pGEX-F:* GTTCCGCGTGGATCCCCGGAATTCATGGATCCACTTCTCGATATACTGAACG  *MaHSFA2c- pGEX-R*: TCAGTCACGATGCGGCCGCTCGAGTCACTTGGGACTAGTGGAACTAAGATAGCCT  *MaBAH1-probe-F:* CAAACATATATGATAAGAAATTTCATTTAACACTCAAGGA  *MaBAH1-probe-R:* TCCTTGAGTGTTAAATGAAATTTCTTATCATATATGTTTG  *MaBAH1-mutant-probe-F:* CAAACATATATGATAATAAATTTAATTTAACACTCAAGGA  *MaBAH1-mutant-probe-R:* TCCTTGAGTGTTAAATTAAATTTATTATCATATATGTTTG | *Eco*R I  *Xho* I |
| **Chip-qPCR** | *MaHSFA2c*-qchip-F: GAAATTTCATTTAACACTCAACGAC  *MaHSFA2c*-qchip-R: CTGAACATATTCTCGGCATTT |  |

**Table S2. Gene codes of HSFs used for phylogenetic analysis.**

| **Name** | **Gene code** |
| --- | --- |
| MaHSFA2c | Ma06_p25790 |
| AtHsfA1a | AT4G17750 |
| AtHsfA1b | AT5G16820 |
| AtHsfA2 | AT2G26150 |
| AtHsfA3 | AT5G03720 |
| AtHsfA4a | AT4G18880 |
| AtHsfA5 | AT4G13980 |
| AtHsfA7a | AT3G51910 |
| AtHsfA8 | AT1G67970 |
| AtHsfA9 | AT5G54070 |
| AtHsfB1 | AT4G36990 |
| AtHsfB2a | AT5G62020 |
| AtHsfB3 | AT2G41690 |
| AtHsfB4 | AT1G46264 |
| OsHsfA1 | LOC_Os03g63750 |
| OsHsfA2a | LOC_Os03g53340 |
| OsHsfA2b | LOC_Os07g08140 |
| OsHsfA2e | LOC_Os03g58160 |
| OsHsfA3 | LOC_Os02g32590 |
| OsHsfA4d | LOC_Os05g45410 |
| OsHsfA5 | LOC_Os02g29340 |
| OsHsfA2d | LOC_Os03g06630 |
| OsHsfA2c | LOC_Os10g28340 |
| OsHsfA6a | LOC_Os06g36930 |
| OsHsfA9 | LOC_Os03g12370 |
| OsHsfB1 | LOC_Os09g28354 |
| OsHsfB2a | LOC_Os04g48030 |
| OsHsfB4a | LOC_Os08g36700 |
| OsHsfC1a | LOC_Os01g43590.1 |
| OsHsfC2a | LOC_Os02g13800 |
